# Angiotensin II therapy in refractory septic shock: which patient can benefit most? A narrative review

**DOI:** 10.1186/s44158-024-00150-w

**Published:** 2024-02-21

**Authors:** Irene Coloretti, Andrea Genovese, J. Pedro Teixeira, Anusha Cherian, Ricard Ferrer, Giovanni Landoni, Marc Leone, Massimo Girardis, Nathan D. Nielsen

**Affiliations:** 1 Anesthesia and Intensive Care Medicine, Policlinico Di Modena, University of Modena and Reggio Emilia, Via del Pozzo, Modena, 71. 41124 Italy; 2 Divisions of Nephrology and Pulmonary, Critical Care, and Sleep Medicine, University of New Mexico School of Medicine, Albuquerque, NM USA; 3 Anesthesiology and Critical Care, Jawaharlal Institute of Postgraduate Medical Education and Research (JIPMER), Dhanvantri Nagar, Pondicherry, India; 4 Intensive Care Department, Hospital Universitari Vall d’Hebron, Barcelona, Spain; 5 Anesthesia and Intensive Care, IRCCS San Raffaele Scientific Institute, Milan, Italy; 6 Anesthesia and Intensive Care Medicine, Hôpital Nord, Assistance Publique Hôpitaux de Marseille, Aix Marseille Université, Marseille, France; 7 Division of Pulmonary, Critical Care and Sleep Medicine & Section of Transfusion Medicine and Therapeutic Pathology, University of New Mexico School of Medicine, Albuquerque, NM USA

**Keywords:** Angiotensin II, Septic shock, Refractory shock, Vasopressors

## Abstract

Patients with septic shock who experience refractory hypotension despite adequate fluid resuscitation and high-dose noradrenaline have high mortality rates. To improve outcomes, evidence-based guidelines recommend starting a second vasopressor, such as vasopressin, if noradrenaline doses exceed 0.5 µg/kg/min. Recently, promising results have been observed in treating refractory hypotension with angiotensin II, which has been shown to increase mean arterial pressure and has been associated with improved outcomes. This narrative review aims to provide an overview of the pathophysiology of the renin-angiotensin system and the role of endogenous angiotensin II in vasodilatory shock with a focus on how angiotensin II treatment impacts clinical outcomes and on identifying the population that may benefit most from its use.

## Background

Septic shock is characterized by hypotension requiring vasopressors to maintain a mean arterial pressure (MAP) of 65mmHg along with elevated serum lactate levels (greater than 2 mmol/L or 18 mg/dL) in the absence of hypovolemia and is associated with profound circulatory, cellular, and metabolic abnormalities [1]. The definition of "refractory septic shock" continues to be a subject of debate. While it has been generally described as "a clinical condition characterized by persistent hyperdynamic shock despite sufficient fluid resuscitation and high doses of noradrenaline (norepinephrine) (at least 1 μg/kg/min)," the exact threshold for vasopressor dosage remains unclear, ranging from 0.2 to 4.0 μg/kg/min [2]. Likewise, the physiological mechanisms underlying refractory septic shock are poorly defined.

Retrospective studies have shown that hypotension is mostly caused by vasodilation and vasoplegia [3]; therefore, vasopressors are the mainstay of hypotension management. In the most recent edition of the Surviving Sepsis Campaign guidelines [4], the use of a vasopressor is suggested in the first hour if the MAP target of 65mmHg is not achieved with fluid resuscitation. Noradrenaline (norepinephrine) is the first-line vasopressor recommended for septic patients and exerts adrenergic activity with marked α1 agonism, moderate β1 agonism, minimal effect on β2 adrenergic receptors, and possesses the ability to increase the cardiac index with a minimal increase in heart rate [4]. However, the use of adrenergic vasopressors is often associated with adverse events, especially at high doses in frail patients. Tachycardia and atrial fibrillation are the most common complications that can impair cardiovascular performance and tissue perfusion. Other side effects include splanchnic and peripheral ischemia as well as derangements of cellular metabolism, including immune cells, leading to lactic acidosis and cellular apoptosis. To reduce these complications, a catecholamine-sparing strategy aimed at minimizing adrenergic vasopressor usage and replacing them with alternative therapies has been recently suggested [4]. This strategy has gained particular interest in the management of refractory shock in which persistent vasoplegia and the desensitization of adrenergic receptors may require the progressive escalation of catecholamines to maintain arterial pressure targets [2].

Evidence-based guidelines suggest initiating a second vasopressor if a high dose of norepinephrine (> 0.5 µg/kg/min) is required [4]. Adrenaline (epinephrine) can be added to noradrenaline (norepinephrine), especially in the presence of cardiac dysfunction or used as a primary vasopressor when its strong β1 effect is desirable. Notably, patients who receive adrenaline (epinephrine) may develop high lactate values not associated with tissue hypoperfusion but rather due to the direct activation of pyruvate dehydrogenase from beta-adrenergic stimulation [5]. The use of vasopressin in conjunction with norepinephrine is a common practice to minimize the negative consequences associated with high levels of catecholamines. This is due to vasopressin's pharmacodynamic properties as an endogenous hormone that acts on V1a, V1b, and V2 receptors and its potential protective effects on renal function and reduction in the occurrence of cardiac arrhythmias [6]. Angiotensin II appears to be a promising and safe non-catecholamine vasopressor as demonstrated by both experimental and human studies [7–9]. These studies, particularly the Angiotensin II for the Treatment of High-Output Shock (ATHOS-3) trial, have shown that angiotensin II increases arterial pressure, leading to its approval as a second-line vasopressor for adults with refractory shock by the Food and Drug Administration (FDA) and European Medicine Agency (EMA) [9].

This narrative review aims to provide a comprehensive overview on the current state of knowledge on the renin-angiotensin system (RAS) as well as the pharmacological effects of, and clinical evidence for, angiotensin II use. Additionally, the review seeks to identify which subgroups of patients with refractory septic shock could benefit the most from this pharmacological therapy.

## Overview of renin-angiotensin system

The RAS is a sophisticated and multi-faceted network of hormones responsible for regulating systemic pressure and fluid and electrolyte balance. Initially, a well-known model depicting a “circulating RAS” was introduced [10]. However, recent studies have indicated that in addition to the “circulating RAS”, a “local RAS” is present in most organs and tissues, characterized by intracellular generation of angiotensin II [11, 12]. Consequently, RAS should be regarded as not only an endocrine system, but also a paracrine and intracrine system. Renin is the initial enzyme in the RAS and is synthesized by pericytes surrounding the kidney afferent arterioles and cells of the juxtaglomerular apparatus where it is stored in intracellular vesicles [13].

Renin release is rapid and is triggered by three stimuli: low blood pressure detected by baroceptors in the afferent arterioles, decreased sodium chloride levels detected by ion transporters in the macula densa of the distal convoluted tubules, or sympathetic nervous system activation through β1 receptors. Its primary function is to cleave angiotensinogen, a pro-hormone synthesized by the liver, to produce angiotensin I. Recent reports described renin as biomarker of severity and tissue perfusion [14, 15], suggesting its important role in homeostasis during critical illness.

Angiotensin-converting enzyme (ACE) then converts angiotensin I into angiotensin II [13]. Alternatively, angiotensin II may be enzymatically generated from angiotensin I by chymase stored in macromolecular complexes in mast cells [11]. This event is possible only in certain pathological conditions as chymase is enzymatically inactive in normal vascular tissue and may produce angiotensin II only in arterial walls [16].

Angiotensin II plays a significant role in maintaining fluid and electrolyte balance as well as regulating hemodynamic and cardiovascular remodelling. It exerts its effects through two distinct receptors, type 1 (AT1) and type 2 (AT2) receptors, with AT1 receptors being responsible for promoting vasoconstriction, the release of vasopressin and aldosterone, stimulating cellular growth and migration, and participating in complex pro-inflammatory processes such as atherosclerosis and vascular aging [17, 18]. The AT2 receptors work in opposition to the AT1 receptors which mediate the effects of bradykinin and nitric oxide to cause vasodilation and usually inhibit cellular growth. However, the clinical significance of these actions is yet to be determined [19].

Angiotensin II plays a crucial role in the cardiovascular system by activating various cellular mechanisms within seconds to minutes, leading to vascular smooth muscle contraction and maintenance of vascular tone [20]. In preclinical models, angiotensin II has been shown to increase myocardial activity through enhanced inotropy and chronotropy, though such effects have not been replicated in humans in which the increased systemic vascular resistance predominates [20].

Additionally, angiotensin II stimulates the synthesis and release of aldosterone, which increases renal sodium absorption and results in higher circulating blood volume. Furthermore, angiotensin II maintains sympathetic outflow to the vasculature and plays a role in the autoregulation of cerebral blood flow. With longer exposure, angiotensin II also promotes metabolic actions such as pro-inflammatory modulation [21], increased insulin secretion [22], B-cell apoptosis [23], and reduction of gluconeogenesis and hepatic glucose output [24]. Angiotensin II is metabolized primarily by end terminal cleavage (at both the amino and carboxyl termini) in erythrocytes, plasma, and many different organs and tissues. This process generates the metabolites angiotensin (1–7) and angiotensin (2–8) (angiotensin III); the true effects of these metabolites are still unclear [13] (Table 1).

**Table 1:** Effects of Angiotensin II, experimental models, and possible clinical relevance in patients with shock

Tissue	Effects	Experimental models	Clinical relevance
Vascular	a) Peripheral vasoconstriction b) Enhanced vascular permeability	Animal Human	^d^
Cardiac	a) Modulation of cardiac remodelling	Animal	^a^
Renal	a) Stimulation of sodium reabsorption in the proximal tubule b) Stimulation of release of aldosterone c) Modulation of renal blood flow	Animal Human	^**c**^
Coagulation	a) Prothrombotic action through stimulation of platelet and plasminogen activator inhibitor-1	In vitro	^b^
Nervous	a) Enhancement of norephrine secretion	Animal	^**a**^
Immune	a) Increase of production of inflammatory mediators (adhesion molecules, cytokines) b) Generation of reactive oxygen species	Animal	^**b**^
Endocrine	a) Stimulation of vasopressin secretion from posterior pituitary gland b) Stimulation of adrenocorticotropin release from anterior pituitary gland c) Enhancement of norepinephrine release via direct action on sympathetic fibres	Animal	^**b**^

^a^Minimal

^b^Valuable

^c^Important

^d^Relevant

## Angiotensin II therapy: clinical evidence

### Sepsis

The therapeutic potential of angiotensin II as an exogenous vasopressor has been known for decades, with its use being described in over a dozen case reports and case series dating to the 1960s [25–37]. However, the agent was not developed for widespread use until after the 2017 ATHOS-3 trial [9]. ATHOS-3 was carried out with input from the FDA and ultimately led to the regulatory approval of angiotensin II in the U.S. by the FDA in 2017 and by the EMA in 2019.

ATHOS-3 was a large (*n* = 344), multicenter, multinational, randomized, blinded, placebo-controlled trial which demonstrated that angiotensin II effectively increased MAP and reduced the need for other vasopressor agents. Were enrolled patients with vasodilatory shock and without cardiac failure. Angiotensin II was initiated when norepinephrine reached doses of 0,2 mcg/kg/min at a dose of 20 ng/kg/min and was subsequently modified every 5 to 15 min for target MAP of 65–75 mmHg. It was observed that with the starting dose over 50% of patients attained the target MAP within 5 min. In contrast, none of the patients who received doses exceeding 80 ng/kg/min (the highest permissible dose) were able to achieve the desired MAP within the first 3 h of treatment. The overall rates of serious adverse effects were similar in both groups (60.7% and 67.1% in the angiotensin II and placebo arms, respectively). Furthermore, though ATHOS-3 was not powered for mortality, a non-significant trend towards decreased 28-day mortality was observed in the angiotensin II arm (46% vs. 54%, *p* = 0.12). The above data resulted in the recommendation for the use of angiotensin II as a third-line vasopressor agent for vasodilatory shock in patients with persistent hypotension despite noradrenaline doses of 0.2–0.3 µg/kg/min and fixed-dose vasopressin, ideally early in the course of shock [38]. Such an approach is consistent with a “multimodal” vasopressor strategy, in which low-to-moderate doses of mechanistically distinct vasopressors are used early with the theoretical benefit of minimizing the toxicity associated with high-dose catecholamine use while also allowing for the clinical detection of responsiveness to individual vasopressor agents [38–43]. This theory is somewhat supported by the consistent (though inherently confounded) observation that high-dose catecholamine exposure is associated with high mortality in patients with sepsis and other types of shock [44–48]. Though a multimodal approach to vasopressor therapy is conceptually appealing, robust prospective data to validate such an approach thus far are lacking. For these reason, these lack of evidence led the Surviving Sepsis Campaign guidelines recommending against first-line use of angiotensin II in septic shock due to insufficient data but do state that angiotensin II may have a role as an adjunctive vasopressor agent due to its demonstrated physiological effectiveness [4].

Notably, though the vast majority of studies of angiotensin II have employed it as a second- or third-line agent, two recent pilot studies reported that angiotensin II as first-line vasopressor may be safe and effective [49, 50]. A further prospective observational study of 40 patients with vasodilatory shock treated initially with angiotensin II compared to 80 matched controls found that angiotensin II was associated with lower ICU mortality and lower rates of troponin elevation, though no adjustment was made for multiple comparisons and no benefit was found in the primary study outcome of peak serum creatinine [49]. Clearly, additional data on the use of angiotensin II as a first-line vasopressor are needed.

### Non-sepsis

While 90% of the patients in ATHOS-3 had confirmed or presumed sepsis, over 5% had postoperative vasoplegia as the cause of vasodilatory shock [9]. A post-hoc analysis of 16 ATHOS-3 subjects with vasoplegia after cardiopulmonary bypass (CPB) demonstrated that angiotensin II was more effective than standard-of-care vasopressors in achieving BP goals [51]. Regarding the use of angiotensin II in cardiac surgery, a feasibility RCT randomized 60 patients to blinded equipotent infusions of angiotensin II or noradrenaline (norepinephrine) which were started after induction of anesthesia prior to initiation of CPB and continued postoperatively as needed [50]. None of the outcomes were statistically significant, but authors found that patients in the angiotensin II group were more likely to achieve BP goals, had a shorter duration of vasopressor need, and had lower rates of acute kidney injury (AKI) and need for renal replacement therapy (RRT). However, these findings are only hypotheses-generating and they should be assessed as part of anappropriately powered studies. Other data describing angiotensin II use for cardiothoracic surgery are limited to case reports, small case series, and subgroups of larger observational studies [42, 52–57], with a few reports in cardiac transplant [58, 59] and extracorporeal membrane oxygenation cases [60]. Notably, multiple human studies have found that exposure to CPB can trigger renin elevation, that the degree of hyperreninemia is associated with the severity of vasoplegia after cardiac surgery, and that angiotensin II use results in decreased renin levels [56, 61–64]. However, despite a mechanistic rationale for angiotensin II use for vasoplegia after CPB, caution is likely warranted in patients with postoperative cardiac dysfunction as decreased cardiac output was an exclusion criterion for angiotensin II in the ATHOS-3 trial [9] and, barring new prospective data demonstrating safety and efficacy, angiotensin II should be avoided in patients with predominantly cardiogenic shock. Overall, though advocated by some [65], additional data are needed prior to recommending the routine use of angiotensin II in cardiac surgery.

## Angiotensin II therapy: safety

The safety profile of angiotensin II appears similar to other vasopressors. A systematic review of > 1,000 studies including > 31,000 patients receiving angiotensin II found a low rate of complications, with the most common serious effects being the worsening of asthma or congestive heart failure [66]. Though overall rates of adverse events were similar in the two arms of ATHOS-3, a numerically high rate (4.3% vs. 0%) of deep venous thrombosis was observed [9]. This observation is consistent with preclinical data suggesting that angiotensin II may be prothrombotic [67, 68]. However, the clinical significance of these observations remains unclear: while a meta-analysis of > 1,700 critically ill patients unexposed to angiotensin II reported a deep vein thrombosis (DVT) rate of 12.7% [69], subsequent observational studies of angiotensin II use reported rates of DVT of ≤ 5% [49, 52, 70]. Nonetheless, pharmacologic DVT prophylaxis is prudent in patients treated with angiotensin II whenever possible.

## Angiotensin II therapy: which patients benefit the most

**Fig. 1:**
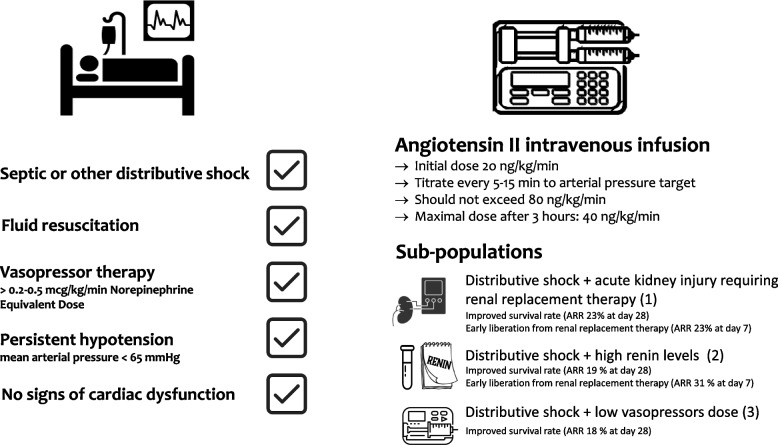
Clinical indications for considering the use of Angiotensin II in critically ill patients with septic shock or other distributive shock (left). Dose and clinical effects in specific sub-populations of Angiotensin II therapy (right)

Despite clinical evidence suggesting that angiotensin II is a safe and effective second- or third-line agent to treat vasodilatory shock, additional prospective data are still needed to determine which patients are most likely to benefit from angiotensin II therapy. As we await additional randomized controlled trials, some data to guide the clinical use of angiotensin II can be gleaned from ATHOS-3 subgroup analyses and observational post-approval studies (Table 2 and Fig. 1).

**Table 2:** Number of patients, sub-populations, outcomes and main limitations of the secondary analysis of ATHOS-3 clinical trial

Title	Patients	Population	Primary outcomes	Other Results	Main Limitiations
**Outcomes in Patients with Vasodilatory Shock and Renal Replacement Therapy Treated with Intravenous Angiotensin II** (Tumlin JA et al. Crit Care Med 2018)	105	Subgroups of ATHOS-3, patients who need RRT at the time of study drug initiation, Placebo (*n* = 60) Ang II ( *n* = 45)	Difference in 28-day survival between Ang II and placebo groups was 53% vs 30% respectively; unadjusted hazard ratio 0.52; 95% CI, [0.30–0.87] (*p* = 0.012)	Day 7 alive and ventilator-free between Ang II and placebo groups was 30,2% vs 10,7% respectively; hazard ratio 3.14 [1.19–8.26] (*p* value = 0.015) Day 7 alive and renal replacement therapy-free between Ang II and placebo groups was 38% vs 15% respectively; hazard ratio, 2.90 [1.29–6.52] (*p* value = 0.007)	Volume status was not analyzed in this post hoc analysis, and the absence of a premorbid serum creatinine precludes any analysis of the impact of preexisting chronic kidney disease
**Outcomes in patients with acute respiratory distress syndrome receiving angiotensin II for vasodilatory shock** (Busse L et al. Critical Care 2018)	321	Subgroup of ATHOS-3, patients with moderate and severe acute ARDS. Placebo *n* = 158: 32% mild ARDS, 32% moderate ARDS, 12% severe ARDS); Ang II *n* = 163: 34% mild ARDS, 30% moderate ARDS, 11% severe ARDS	Mean arterial pressure response was achieved by 24%, 25%, and 16% of patients in the placebo group and 67% (OR = 6.7, *p* < 0.001), 69% (OR = 6.6, *p* < 0.001), and 61% (OR = 8.4, *p* = 0.005) in the Ang II group for mild, moderate and severe ARDS respectively	28-day mortality between placebo and Ang II groups related to ARDS severity. Placebo: Mild 53 [40–67] Moderate 55 [42–69] Severe 74 [53—90]; Ang II Mild 42 [30–56] Moderate 55 [42–69] Severe 50 [30–74]	NA
**Renin and Survival in Patients Given Angiotensin II for Catecholamine-Resistant Vasodilatory Shock: A Clinical Trial.** (Bellomo R et al. Am J Respir Crit Care Med. 2020)	255	Subgroup of ATHOS-3, patients with serum samples available for analysis of renin concentrations at baseline Renin below the median population value (< 172.7 pg/ml) *n* = 127, placebo 63; Ang II 64; Renin above the median population value (> 172.7 pg/ml) *n* = 128. placebo 73; Ang II 55)	28-day mortality rate between placebo and Ang II in patients with renin concentration above the median was 69.9% vs 51.1% respectively, hazard ratio, 0.556 [95% CI, 0.35–0.88] (*p* = 0.0115). There was no statistically significant difference in 28-day mortality for those with serum renin concentrations below median	In patients with renin concentration above the median the rate of renal replacement therapy liberation by Day 7 was: Ang II 43% vs placebo 12% (*p* = 0.01). The mean change in cardiovascular SOFA score at 48 h was significantly greater in the Ang II group (- 1.56 ± 1.79) vs placebo group (- 0.78 ± 1.39) There was no statistically significant difference for those with serum renin concentrations below median	No measure of angiotensin 1–7, bradykinin, or aldosterone concentrations,. No rigorously assess for the presence or severity of preexisting hypertension, renovascular disease, or chronic kidney disease
**Initiating angiotensin II at lower vasopressor** **doses in vasodilatory shock: an exploratory** **post-hoc analysis of the ATHOS-3 clinical trial** (Wieruszewski et al. Crit Care 2023)	321	Subgroups of ATHOS-3, Low NED (≤ 0.25 μg/kg/min; *n* = 104. placebo 48; ANGII 56); High NED (> 0.25 μg/kg/min; *n* = 217. placebo 110; ANGII 107)	Difference in 28-day survival between the Ang II and placebo in NED ≤ 0.25 μg/kg/min: 64% vs 48% (p 0.03); NED > 0.25 μg/kg/min: 49% vs 45%; (p 0.71)	Difference in incidence of discontinuing RRT at day 7 between Ang II and placebo in NED ≤ 0.25 μg/kg/min 59% vs 33% (*p* = 0.29); NED > 0.25 μg/kg/min 29% vs 11% (*p* = 0.03)	The study design resulted in only one third of patients being enrolled at NED ≤ 0.25 μg/ kg/min, limiting the generalizability of these data. It is possible that low-NED and high-NED do not correlate with early or late stage of disease

Ang II: angiotensin II; *ARDS* Acute respiratory distress syndrome, *NED* norepinephrine dose equivalent

In a prespecified subgroup analysis of the sickest patients in ATHOS-3 (i.e.: those with APACHE II scores > 30), the angiotensin II arm had a lower 28-day mortality compared to placebo (51.8% vs. 70.8%,* p* = 0.037) [71]. On the other hand, a post-hoc analysis of ATHOS-3 found decreased 28-day mortality among the 104 patients on noradrenaline (norepinephrine) equivalent doses (NED) of vasopressors of ≤ 0.25 µg/kg/min at study drug initiation (adjusted hazard ratio 0.51, *p* = 0.03) [72], suggesting that *earlier* initiation of angiotensin II may be beneficial. The finding that angiotensin II appears most helpful earlier in the course of shock is supported by observational data demonstrating enhanced BP benefit when angiotensin II is started at NED of < 0.2 or < 0.3 µg/kg/min or when introduced as a second- or third-line vasopressor agent (rather than fourth- or fifth-line) [52]. Similarly, another observational study suggests that angiotensin II is less effective when initiated late, i.e.: when NED is > 1 µg/kg/min [73].

Otherwise, both observational data and mechanistic studies suggest that angiotensin II may be particularly effective in patients with AKI, acute hypoxemic respiratory failure, and high-renin shock states. In another post-hoc subgroup analysis of ATHOS-3 of patients with AKI requiring RRT at randomization, angiotensin II was associated with statistically significant benefits in both mortality and liberation from RRT [74], an effect felt to reflect angiotensin II’s ability to increase or preserve glomerular filtration through preferential vasoconstriction of the efferent renal arteriole. Yet another post-hoc analysis of ATHOS-3 suggested benefit in patients with combined shock and ARDS with a non-statistically significant trend towards improved mortality [75]. Several subsequent observational studies found that treatment with angiotensin II was associated with improved oxygenation or a decreased need for oxygenation support (fraction of inspired oxygen or positive end-expiratory pressure) in patients with respiratory failure from COVID-19 [76–78] or other causes [79]. A benefit from angiotensin II in ARDS is biologically plausible given that ACE is present at high levels in the pulmonary vascular endothelium, and therefore ARDS patients with septic shock may be particularly deficient in angiotensin II. Likewise, in a post-hoc ATHOS-3 subgroup analysis, angiotensin II use was associated with a statistically significant reduction in 28-day mortality in 128 subjects with renin concentrations above the median (50.9% vs. 69.9%, *p* = 0.012) [61]. Subsequent observational data suggest that renin levels may also be used to monitor response to angiotensin II therapy [80].

## Conclusions

Patients with septic shock, especially those with persistent hypotension despite fluid resuscitation and a robust dose of vasopressors, remain challenging, with high mortality risk and limited evidence to guide appropriate management. A number of clinical studies have highlighted the limitations and potential hazards associated with the use of elevated doses of catecholamines. As a result, it is commonly advised to employ a combination of various vasoactive medications in cases of refractory shock. Based on both its pharmacological properties and accumulating clinical evidence, angiotensin II can be considered a useful option as a secondary or tertiary vasopressor to restore an appropriate MAP in patients with persistent shock. The trial data available, reinforced by the role of endogenous angiotensin II in regulating renal blood flow, suggest that therapy with pharmacologic angiotensin II may be beneficial for patients with shock and AKI. Similarly, the correlation between endogenous angiotensin II levels and survival rates, as well as the inverse relationship between angiotensin II and plasma renin levels, suggest that angiotensin II therapy may be advantageous for patients with shock and elevated renin levels. However, it should be noted, that all these findings were derived from secondary analyses of a single clinical trial. Therefore, future trials should be conducted to verify these hypotheses to better identify which patients can benefit the most from angiotensin II therapy.

## Data Availability

Data sharing is not applicable to this article as no datasets were generated or analysed during the current study.

## Abbreviations

RAS: Renin-Angiotensin system
ACE: Angiotensin-converting enzyme
AT1: Angiotensin II receptor type 1
AT2: Angiotensin II receptor type 2
U.S: United States
FDA: Food and Drug Administration
MAP: Mean arterial pressure
BP: Blood pressure
CBP: Cardiopulmonary bypass
AKI: Acute kidney injury
RRT: Renal replacement therapy
DVT: Deep vein thrombosis
RCT: Randomized-controlled trial
APACHE: Acute Physiology and Chronic Health Evaluation
NED: Noradrenaline equivalent doses
ARDS: Acute respiratory distress syndrome

## References

[1] Singer M, Deutschman CS, Seymour CW, Shankar-Hari M, Annane D, Bauer M et al (2016) The third international consensus definitions for sepsis and septic shock (Sepsis-3). JAMA 315:801–810. 10.1001/jama.2016.0287 26903338 10.1001/jama.2016.0287 PMC4968574

[2] Antonucci E, Polo T, Giovini M, Girardis M, Martin-Loeches I, Nielsen ND et al (2023) Refractory septic shock and alternative wordings: a systematic review of literature. J Crit Care 75:154258. 10.1016/j.jcrc.2023.154258 36706554 10.1016/j.jcrc.2023.154258

[3] Maheshwari K, Nathanson BH, Munson SH, Khangulov V, Stevens M, Badani H et al (2018) The relationship between ICU hypotension and in-hospital mortality and morbidity in septic patients. Intensive Care Med 44:857–867. 10.1007/s00134-018-5218-5 29872882 10.1007/s00134-018-5218-5 PMC6013508

[4] Evans L, Rhodes A, Alhazzani W, Antonelli M, Coopersmith CM, French C et al (2021) Surviving sepsis campaign: international guidelines for management of sepsis and septic shock 2021. Crit Care Med 49:e1063–e1143. 10.1097/CCM.0000000000005337 34605781 10.1097/CCM.0000000000005337

[5] Myburgh JA, Higgins A, Jovanovska A, Lipman J, Ramakrishnan N, Santamaria J et al (2008) A comparison of epinephrine and norepinephrine in critically ill patients. Intensive Care Med 34:2226–2234. 10.1007/s00134-008-1219-0 18654759 10.1007/s00134-008-1219-0

[6] Barabutis N, Marinova M, Solopov P, Uddin MA, Croston GE, Reinheimer TM et al (2020) Protective Mechanism of the Selective Vasopressin V1A Receptor Agonist Selepressin against Endothelial Barrier Dysfunction. J Pharmacol Exp Ther 375:286–295. 10.1124/jpet.120.000146 32943478 10.1124/jpet.120.000146

[7] Chawla LS, Busse LW, Brasha-Mitchell E, Alotaibi Z (2016) The use of angiotensin II in distributive shock. Crit Care 20:137. 10.1186/s13054-016-1306-5 27230465 10.1186/s13054-016-1306-5 PMC4882778

[8] Corrêa TD, Jeger V, Pereira AJ, Takala J, Djafarzadeh S, Jakob SM (2014) Angiotensin II in septic shock: effects on tissue perfusion, organ function, and mitochondrial respiration in a porcine model of fecal peritonitis. Crit Care Med 42:e550-559. 10.1097/CCM.0000000000000397 24797374 10.1097/CCM.0000000000000397

[9] Khanna A, English SW, Wang XS, Ham K, Tumlin J, Szerlip H et al (2017) Angiotensin II for the Treatment of Vasodilatory Shock. N Engl J Med 377:419–430. 10.1056/NEJMoa1704154 28528561 10.1056/NEJMoa1704154

[10] Ichihara A, Kobori H, Nishiyama A, Navar LG (2004) Renal renin-angiotensin system. Contrib Nephrol 143:117–130. 10.1159/000078716 15248360 10.1159/000078716 PMC2575669

[11] Fyhrquist F, Saijonmaa O (2008) Renin-angiotensin system revisited. J Intern Med 264:224–236. 10.1111/j.1365-2796.2008.01981.x 18793332 10.1111/j.1365-2796.2008.01981.x PMC7166930

[12] Kumar R, Singh VP, Baker KM (2007) The intracellular renin-angiotensin system: a new paradigm. Trends Endocrinol Metab 18:208–214. 10.1016/j.tem.2007.05.001 17509892 10.1016/j.tem.2007.05.001

[13] Paul M, Poyan Mehr A, Kreutz R (2006) Physiology of local renin-angiotensin systems. Physiol Rev 86:747–803. 10.1152/physrev.00036.2005 16816138 10.1152/physrev.00036.2005

[14] Jeyaraju M, McCurdy MT, Levine AR, Devarajan P, Mazzeffi MA, Mullins KE et al (2022) Renin kinetics are superior to lactate kinetics for predicting in-hospital mortality in hypotensive critically Ill patients. Crit Care Med 50:50–60. 10.1097/CCM.0000000000005143 34166293 10.1097/CCM.0000000000005143

[15] Gleeson PJ, Crippa IA, Mongkolpun W, Cavicchi FZ, Van Meerhaeghe T, Brimioulle S et al (2019) Renin as a marker of tissue-perfusion and prognosis in critically Ill patients. Crit Care Med 47:152–158. 10.1097/CCM.0000000000003544 30653055 10.1097/CCM.0000000000003544

[16] Kokkonen JO, Lindstedt KA, Kovanen PT (2003) Role for chymase in heart failure: angiotensin II-dependent or -independent mechanisms? Circulation 107:2522–2524. 10.1161/01.CIR.0000074786.92067.AA 12777313 10.1161/01.CIR.0000074786.92067.AA

[17] Mehta PK, Griendling KK (2007) Angiotensin II cell signaling: physiological and pathological effects in the cardiovascular system. Am J Physiol Cell Physiol 292:C82-97. 10.1152/ajpcell.00287.2006 16870827 10.1152/ajpcell.00287.2006

[18] Fleming I, Kohlstedt K, Busse R (2006) The tissue renin-angiotensin system and intracellular signalling. Curr Opin Nephrol Hypertens 15:8–13. 10.1097/01.mnh.0000196146.65330.ea 16340660 10.1097/01.mnh.0000196146.65330.ea

[19] Carey RM (2005) Update on the role of the AT2 receptor. Curr Opin Nephrol Hypertens 14:67–71. 10.1097/00041552-200501000-00011 15586018 10.1097/00041552-200501000-00011

[20] Baker KM, Booz GW, Dostal DE (1992) Cardiac actions of angiotensin II: Role of an intracardiac renin-angiotensin system. Annu Rev Physiol 54:227–241. 10.1146/annurev.ph.54.030192.001303 1562174 10.1146/annurev.ph.54.030192.001303

[21] Ruiz-Ortega M, Ruperez M, Lorenzo O, Esteban V, Blanco J, Mezzano S, et al. (2002) Angiotensin II regulates the synthesis of proinflammatory cytokines and chemokines in the kidney. Kidney Int Suppl S12–22 10.1046/j.1523-1755.62.s82.4.x 10.1046/j.1523-1755.62.s82.4.x 12410849

[22] Ramracheya RD, Muller DS, Wu Y, Whitehouse BJ, Huang GC, Amiel SA et al (2006) Direct regulation of insulin secretion by angiotensin II in human islets of Langerhans. Diabetologia 49:321–331. 10.1007/s00125-005-0101-7 16416272 10.1007/s00125-005-0101-7

[23] Chu KY, Lau T, Carlsson P-O, Leung PS (2006) Angiotensin II type 1 receptor blockade improves beta-cell function and glucose tolerance in a mouse model of type 2 diabetes. Diabetes 55:367–374. 10.2337/diabetes.55.02.06.db05-1022 16443769 10.2337/diabetes.55.02.06.db05-1022

[24] Assimacopoulos-Jeannet FD, Blackmore PF, Exton JH (1982) Studies of the interaction between glucagon and alpha-adrenergic agonists in the control of hepatic glucose output. J Biol Chem 257:3759–3765 6120943

[25] Del Greco F, Johnson DC (1961) Clinical experience with angiotensin II in the treatment of shock. JAMA 178:994–999. 10.1001/jama.1961.03040490020005 13884972 10.1001/jama.1961.03040490020005

[26] Derrick JR, Anderson JR, Roland BJ (1962) Adjunctive use of a biologic pressor agent, angiotensin, in management of shock. Circulation 25:263–267. 10.1161/01.cir.25.1.263 13885617 10.1161/01.cir.25.1.263

[27] Wedeen R, Zucker G (1963) Angiotensin II in the treatment of shock. Am J Cardiol 11:82–86. 10.1016/0002-9149(63)90036-5 13999441 10.1016/0002-9149(63)90036-5

[28] Singh S, Malhotra RP (1966) Comparative study of angiotensin and nor-adrenaline in hypotensive states (shock). J Assoc Physicians India 14:639–645 4292372

[29] Thomas VL, Nielsen MS (1991) Administration of angiotensin II in refractory septic shock. Crit Care Med 19:1084–1086. 10.1097/00003246-199108000-00020 1860334 10.1097/00003246-199108000-00020

[30] Jackson T, Corke C, Agar J (1993) Enalapril overdose treated with angiotensin infusion. Lancet 341:703. 10.1016/0140-6736(93)90479-z 8095618 10.1016/0140-6736(93)90479-z

[31] Lisinopril overdose and management with intravenous angiotensin II - PubMed n.d. https://pubmed.ncbi.nlm.nih.gov/7841571/ (Accessed 24 Nov 2023) 10.1177/106002809402801006 7841571

[32] Newby DE, Lee MR, Gray AJ, Boon NA (1995) Enalapril overdose and the corrective effect of intravenous angiotensin II. Br J Clin Pharmacol 40:103–104. 10.1111/j.1365-2125.1995.tb04546.x 8527259 10.1111/j.1365-2125.1995.tb04546.x PMC1365039

[33] Ryding J, Heslet L, Hartvig T, Jønsson V (1995) Reversal of “refractory septic shock” by infusion of amrinone and angiotensin II in an anthracycline-treated patient. Chest 107:201–203. 10.1378/chest.107.1.201 7813278 10.1378/chest.107.1.201

[34] Wray GM, Coakley JH (1995) Severe septic shock unresponsive to noradrenaline. Lancet 346:1604. 10.1016/s0140-6736(95)91933-3 7500755 10.1016/s0140-6736(95)91933-3

[35] Tovar JL, Bujons I, Ruiz JC, Ibañez L, Salgado A (1997) Treatment of severe combined overdose of calcium antagonists and converting enzyme inhibitors with angiotensin II. Nephron 77:239. 10.1159/000190280 9346394 10.1159/000190280

[36] Desachy A, Normand S, François B, Cassat C, Gastinne H (2000) Vignon P [Refractory shock after converting enzyme inhibitor administration. Usefulness of angiotensin II]. Presse Med 29:696–698 10797820

[37] Yunge M, Petros A (2000) Angiotensin for septic shock unresponsive to noradrenaline. Arch Dis Child 82:388–389. 10.1136/adc.82.5.388 10799431 10.1136/adc.82.5.388 PMC1718307

[38] Ammar MA, Ammar AA, Wieruszewski PM, Bissell BD, T Long M, Albert L et al (2022) Timing of vasoactive agents and corticosteroid initiation in septic shock. Ann Intensive Care 12:47. 10.1186/s13613-022-01021-9 35644899 10.1186/s13613-022-01021-9 PMC9148864

[39] Chawla LS, Ostermann M, Forni L, Tidmarsh GF (2019) Broad spectrum vasopressors: a new approach to the initial management of septic shock? Crit Care 23:124. 10.1186/s13054-019-2420-y 30992045 10.1186/s13054-019-2420-y PMC6469125

[40] Wakefield BJ, Sacha GL, Khanna AK (2018) Vasodilatory shock in the ICU and the role of angiotensin II. Curr Opin Crit Care 24:277–285. 10.1097/MCC.0000000000000517 29877879 10.1097/MCC.0000000000000517

[41] Zhong L, Ji X-W, Wang H-L, Zhao G-M, Zhou Q, Xie B (2020) Non-catecholamine vasopressors in the treatment of adult patients with septic shock-evidence from meta-analysis and trial sequential analysis of randomized clinical trials. J Intensive Care 8:83. 10.1186/s40560-020-00500-0 33292658 10.1186/s40560-020-00500-0 PMC7603734

[42] Wieruszewski PM, Khanna AK (2022) Vasopressor Choice and Timing in Vasodilatory Shock. Crit Care 26:76. 10.1186/s13054-022-03911-7 35337346 10.1186/s13054-022-03911-7 PMC8957156

[43] Leone M, Einav S, Antonucci E, Depret F, Lakbar I, Martin-Loeches I et al (2023) Multimodal strategy to counteract vasodilation in septic shock. Anaesth Crit Care Pain Med 42:101193. 10.1016/j.accpm.2023.101193 36621622 10.1016/j.accpm.2023.101193

[44] Auchet T, Regnier M-A, Girerd N, Levy B (2017) Outcome of patients with septic shock and high-dose vasopressor therapy. Ann Intensive Care 7:43. 10.1186/s13613-017-0261-x 28425079 10.1186/s13613-017-0261-x PMC5397393

[45] Brown SM, Lanspa MJ, Jones JP, Kuttler KG, Li Y, Carlson R et al (2013) Survival after shock requiring high-dose vasopressor therapy. Chest 143:664–671. 10.1378/chest.12-1106 22911566 10.1378/chest.12-1106 PMC3590882

[46] Roberts RJ, Miano TA, Hammond DA, Patel GP, Chen J-T, Phillips KM et al (2020) Evaluation of Vasopressor Exposure and Mortality in Patients With Septic Shock. Crit Care Med 48:1445–1453. 10.1097/CCM.0000000000004476 32706559 10.1097/CCM.0000000000004476

[47] Sato R, Duggal A, Sacha GL, Rudoni MA, Yataco AC, Khanna AK et al (2023) The relationship between norepinephrine equivalent dose of vasopressors within 24 hours from the onset of septic shock and in-hospital mortality Rate. Chest 163:148–151. 10.1016/j.chest.2022.07.018 35921884 10.1016/j.chest.2022.07.018

[48] Sviri S, Hashoul J, Stav I, van Heerden PV (2014) Does high-dose vasopressor therapy in medical intensive care patients indicate what we already suspect? J Crit Care 29:157–160. 10.1016/j.jcrc.2013.09.004 24140297 10.1016/j.jcrc.2013.09.004

[49] See EJ, Clapham C, Liu J, Khasin M, Liskaser G, Chan JW et al (2023) A pilot study of angiotensin ii as primary vasopressor in critically ill adults with vasodilatory hypotension: the Aramis study. Shock 59:691–696. 10.1097/SHK.0000000000002109 36930693 10.1097/SHK.0000000000002109

[50] Coulson TG, Miles LF, Serpa Neto A, Pilcher D, Weinberg L, Landoni G et al (2022) A double-blind randomised feasibility trial of angiotensin-2 in cardiac surgery. Anaesthesia 77:999–1009. 10.1111/anae.15802 35915923 10.1111/anae.15802 PMC9543254

[51] Klijian A, Khanna AK, Reddy VS, Friedman B, Ortoleva J, Evans AS et al (2021) Treatment With Angiotensin II Is Associated With Rapid Blood Pressure Response and Vasopressor Sparing in Patients With Vasoplegia After Cardiac Surgery: A Post-Hoc Analysis of Angiotensin II for the Treatment of High-Output Shock (ATHOS-3) Study. J Cardiothorac Vasc Anesth 35:51–58. 10.1053/j.jvca.2020.08.001 32868152 10.1053/j.jvca.2020.08.001

[52] Smith SE, Newsome AS, Guo Y, Hecht J, McCurdy MT, Mazzeffi MA et al (2022) A multicenter observational cohort study of angiotensin ii in shock. J Intensive Care Med 37:75–82. 10.1177/0885066620972943 33231111 10.1177/0885066620972943 PMC8559525

[53] Bird S, Chand M, Tran TL, Ali S, Awad SS, Cornwell LD et al (2023) Evaluation of the addition of angiotensin II in patients with shock after cardiac surgery at a veterans affairs medical center. Ann Pharmacother 57:141–147. 10.1177/10600280221099928 35658717 10.1177/10600280221099928

[54] Evans A, McCurdy MT, Weiner M, Zaku B, Chow JH (2019) Use of angiotensin II for post cardiopulmonary Bypass Vasoplegic Syndrome. Ann Thorac Surg 108:e5-7. 10.1016/j.athoracsur.2018.11.047 30582924 10.1016/j.athoracsur.2018.11.047

[55] Konkol SB, Morrisette MJ, Hulse MC, Enfield KB, Mihalek AD (2022) Outcomes following the use of angiotensin II in patients with postoperative vasoplegic syndrome: a case series. Ann Card Anaesth 25:359–361. 10.4103/aca.aca_98_21 35799569 10.4103/aca.aca_98_21 PMC9387627

[56] Trethowan B, Michaud CJ, Fifer S (2020) Use of angiotensin II in severe vasoplegia after left pneumonectomy requiring cardiopulmonary bypass: a renin response analysis. Crit Care Med 48:e912–e915. 10.1097/CCM.0000000000004502 32931196 10.1097/CCM.0000000000004502

[57] Wieruszewski PM, Radosevich MA, Kashani KB, Daly RC, Wittwer ED (2019) Synthetic human angiotensin II for postcardiopulmonary bypass vasoplegic shock. J Cardiothorac Vasc Anesth 33:3080–3084. 10.1053/j.jvca.2019.03.004 31060933 10.1053/j.jvca.2019.03.004

[58] Cutler NS, Rasmussen BM, Bredeck JF, Lata AL, Khanna AK (2021) Angiotensin II for critically ill patients with shock after heart transplant. J Cardiothorac Vasc Anesth 35:2756–2762. 10.1053/j.jvca.2020.07.087 32868151 10.1053/j.jvca.2020.07.087

[59] Wieruszewski PM, Sims CR, Daly RC, Taner T, Wittwer ED (2019) Use of angiotensin II for vasoplegic shock in a combined heart and liver transplant recipient with systolic anterior motion physiology. J Cardiothorac Vasc Anesth 33:2366–2367. 10.1053/j.jvca.2019.03.054 31076311 10.1053/j.jvca.2019.03.054

[60] Ostermann M, Boldt DW, Harper MD, Lim GW, Gunnerson K (2018) Angiotensin in ECMO patients with refractory shock. Crit Care 22:288. 10.1186/s13054-018-2225-4 30382926 10.1186/s13054-018-2225-4 PMC6211436

[61] Meersch M, Weiss R, Massoth C, Küllmar M, Saadat-Gilani K, Busen M et al (2022) The association between angiotensin ii and renin kinetics in patients after cardiac surgery. Anesth Analg 134:1002–1009. 10.1213/ANE.0000000000005953 35171852 10.1213/ANE.0000000000005953

[62] Montgomery ML, Gross CR, Lin H-M, Ouyang Y, Levin MA, Corkill HE et al (2023) Plasma Renin Activity Increases With Cardiopulmonary Bypass and is Associated With Vasoplegia After Cardiac Surgery. J Cardiothorac Vasc Anesth 37:367–373. 10.1053/j.jvca.2022.11.019 36509636 10.1053/j.jvca.2022.11.019

[63] Coulson TG, Miles LF, Zarbock A, Burrell LM, Patel SK, von Groote T et al (2023) Renin-angiotensin-aldosterone system dynamics after targeted blood pressure control using angiotensin II or norepinephrine in cardiac surgery: mechanistic randomised controlled trial. Br J Anaesth 131:664–672. 10.1016/j.bja.2023.06.056 37481435 10.1016/j.bja.2023.06.056

[64] Küllmar M, Saadat-Gilani K, Weiss R, Massoth C, Lagan A, Cortés MN et al (2021) Kinetic changes of plasma renin concentrations predict acute kidney injury in cardiac surgery patients. Am J Respir Crit Care Med 203:1119–1126. 10.1164/rccm.202005-2050OC 33320784 10.1164/rccm.202005-2050OC

[65] Chow JH, Wittwer ED, Wieruszewski PM, Khanna AK (2022) Evaluating the evidence for angiotensin II for the treatment of vasoplegia in critically ill cardiothoracic surgery patients. J Thorac Cardiovasc Surg 163:1407–1414. 10.1016/j.jtcvs.2021.02.097 33875258 10.1016/j.jtcvs.2021.02.097

[66] Busse LW, Barker N, Petersen C (2020) Vasoplegic syndrome following cardiothoracic surgery-review of pathophysiology and update of treatment options. Crit Care 24:36. 10.1186/s13054-020-2743-8 32019600 10.1186/s13054-020-2743-8 PMC7001322

[67] Senchenkova EY, Russell J, Almeida-Paula LD, Harding JW, Granger DN (2010) Angiotensin II-mediated microvascular thrombosis. Hypertension 56:1089–1095. 10.1161/HYPERTENSIONAHA.110.158220 20975035 10.1161/HYPERTENSIONAHA.110.158220 PMC3023299

[68] Mogielnicki A, Chabielska E, Pawlak R, Szemraj J, Buczko W (2005) Angiotensin II enhances thrombosis development in renovascular hypertensive rats. Thromb Haemost 93:1069–1076. 10.1160/TH04-10-0701 15968390 10.1160/TH04-10-0701

[69] Malato A, Dentali F, Siragusa S, Fabbiano F, Kagoma Y, Boddi M et al (2015) The impact of deep vein thrombosis in critically ill patients: a meta-analysis of major clinical outcomes. Blood Transfus 13:559–568. 10.2450/2015.0277-14 26513770 10.2450/2015.0277-14 PMC4624530

[70] Wieruszewski PM, Wittwer ED, Kashani KB, Brown DR, Butler SO, Clark AM et al (2021) Angiotensin II infusion for shock: a multicenter study of postmarketing use. Chest 159:596–605. 10.1016/j.chest.2020.08.2074 32882250 10.1016/j.chest.2020.08.2074 PMC7856533

[71] Szerlip H, Bihorac A, Chang S, Chung K, Hästbacka J, Murugan R et al (2018) 6: effect of disease severity on survival in patients receiving angiotensin ii for vasodilatory shock. Crit Care Med 46:3. 10.1097/01.ccm.0000528062.45598.be

[72] Wieruszewski PM, Bellomo R, Busse LW, Ham KR, Zarbock A, Khanna AK et al (2023) Initiating angiotensin II at lower vasopressor doses in vasodilatory shock: an exploratory post-hoc analysis of the ATHOS-3 clinical trial. Crit Care 27:175. 10.1186/s13054-023-04446-1 37147690 10.1186/s13054-023-04446-1 PMC10163684

[73] Quan M, Cho N, Bushell T, Mak J, Nguyen N, Litwak J et al (2022) Effectiveness of angiotensin ii for catecholamine refractory septic or distributive shock on mortality: a propensity score weighted analysis of real-world experience in the medical ICU. Crit Care Explor 4:e0623. 10.1097/CCE.0000000000000623 35072084 10.1097/CCE.0000000000000623 PMC8769135

[74] Tumlin JA, Murugan R, Deane AM, Ostermann M, Busse LW, Ham KR et al (2018) Outcomes in Patients with Vasodilatory Shock and Renal Replacement Therapy Treated with Intravenous Angiotensin II. Crit Care Med 46:949–957. 10.1097/CCM.0000000000003092 29509568 10.1097/CCM.0000000000003092 PMC5959265

[75] 38th International Symposium on Intensive Care and Emergency Medicine - PMC n.d. https://www.ncbi.nlm.nih.gov/pmc/articles/PMC5883106/ (Accessed 24 Nov 2023)

[76] Zangrillo A, Landoni G, Beretta L, Morselli F, Serpa Neto A, Bellomo R et al (2020) Angiotensin II infusion in COVID-19-associated vasodilatory shock: a case series. Crit Care 24:227. 10.1186/s13054-020-02928-0 32414393 10.1186/s13054-020-02928-0 PMC7228670

[77] Leisman DE, Mastroianni F, Fisler G, Shah S, Hasan Z, Narasimhan M et al (2020) Physiologic response to angiotensin II treatment for coronavirus disease 2019-induced vasodilatory shock: a retrospective matched cohort study. Crit Care Explor 2:e0230. 10.1097/CCE.0000000000000230 33063034 10.1097/CCE.0000000000000230 PMC7523856

[78] Serpa Neto A, Landoni G, Ostermann M, Lumlertgul N, Forni L, Alvarez-Belon L et al (2022) Angiotensin II infusion in COVID-19: an international, multicenter, registry-based study. J Med Virol 94:2079–2088. 10.1002/jmv.27592 35029318 10.1002/jmv.27592 PMC9015246

[79] Bellomo R, Forni LG, Busse LW, McCurdy MT, Ham KR, Boldt DW et al (2020) Renin and survival in patients given angiotensin II for catecholamine-resistant vasodilatory shock. A clinical trial. Am J Respir Crit Care Med 202:1253–61. 10.1164/rccm.201911-2172OC 32609011 10.1164/rccm.201911-2172OC PMC7605187

[80] Busse LW, Wang XS, Chalikonda DM, Finkel KW, Khanna AK, Szerlip HM et al (2017) Clinical experience with IV angiotensin II administration: a systematic review of safety. Crit Care Med 45:1285–1294. 10.1097/CCM.0000000000002441 28489648 10.1097/CCM.0000000000002441 PMC5515638

